# Single particle optical extinction and scattering allows real time quantitative characterization of drug payload and degradation of polymeric nanoparticles

**DOI:** 10.1038/srep18228

**Published:** 2015-12-15

**Authors:** M. A. C. Potenza, T. Sanvito, S. Argentiere, C. Cella, B. Paroli, C. Lenardi, P. Milani

**Affiliations:** 1 CIMAINA and Dipartimento di Fisica, Università degli Studi di Milano, via Celoria 16, 20133 Milano, Italy; 2 EOS srl, viale Ortles 22/4, 20139, Milano, Italy; 3 Fondazione Filarete, viale Ortles 22/4, 20139 Milano, Italy; 4 SEMM, European School of Molecular Medicine, Campus IFOM-IEO, via Adamello 16, 20139 Milano, Italy

## Abstract

The behavior of nanoparticles in biological systems is determined by their dimensions, size distribution, shape, surface chemistry, density, drug loading and stability; the characterization of these parameters in realistic conditions and the possibility to follow their evolution *in vitro* and *in vivo* are, in most of the cases, far from the capabilities of the standard characterization technologies. Optical techniques such as dynamic light scattering (DLS) are, in principle, well suited for in line characterization of nanoparticle, however their fail in characterizing the evolution of nanoparticle in solution where change in particle dimension and density is present. Here we present an in-line optical technique based on single particle extinction and scattering (SPES) overcoming the limitations typical of DLS and allowing for the efficient characterization of nanoparticle polydispersity, index of refraction and degradation dynamics in solution. Using SPES, we characterized the evolution of PLGA nanoparticles with different structures and drug payloads in solution and we compared the results with DLS. Our results suggest that SPES could be used as a process analytical technology for pharmaceutical nanoparticle production.

The use of nanoparticles (NPs) as carriers for cancer therapy is considered a revolutionary improvement enhancing the therapeutic efficacy and reducing adverse effects related to the non-specificity of anti-cancer drugs^1^,^2^,^3^. NPs are expected to provide a superior control on drug pharmacokinetics and bio-distribution, their surface can also be functionalized in order to target with high precision the tumor site^4^. In particular synthetic polymer NPs are subject of particular attention for their capability of responding to external physico-chemical stimuli (temperature, pH, etc.) and modifying their structure in order to release the drug payload in a programmable way^5^,^6^.

Despite the hype surrounding novel therapeutic solutions based on polymeric NPs and witnessed by an impressive number of scientific publications in the last few years, the translation of NPs carriers to clinical trials and real clinical practice is exiguous since major problems about safety, regulatory and manufacturing issues remain unsolved^7^,^8^,^9^.

The behavior of NPs in biological systems is determined by their dimensions, size distribution, shape, surface chemistry, density, drug loading and stability^10^,^11^,^12^; the characterization of these parameters in realistic conditions and the possibility to follow their evolution *in vitro* and *in vivo* are, in most of the cases, far from the capabilities of the standard characterization technologies^11^,^12^,^13^. Design and optimization of NPs formulations able to meet the pharmaceutical standards require measurement systems capable of real time or near real time (e.g., on-, in-, or at-line) monitoring of all critical attributes of the carrier nanoparticles and of their payload^13^. To date, polymeric NPs with high drug-loading capacity and the ability to control the drug release have been reported, however their performances in terms of polymer degradation rate and release kinetics of the entrapped therapeutic agents are characterized with techniques and in conditions not completely suited to support a translation in clinical practice^14^. In particular the encapsulation of both hydrophobic and hydrophilic drugs into polymeric NPs and their subsequent release is mainly characterized by off-line chromatographic and spectroscopic techniques. Noteworthy, although these methods can be applied to assess the encapsulation of drugs into the NPs, they cannot provide precise information about drug-loaded NPs evolution and structural changes in complex biological fluids^15^,^16^,^17^,^18^. The lack of in line characterization techniques reducing batch failures and improving yields also hampers the development of industrial manufacturing methods for NPs.

One of the most popular and routine methods to characterize NPs in solution is Dynamic Light Scattering (DLS); although very straightforward and easy-to-use, it cannot give information about the NPs structure but only on their hydrodynamic radius^12^,^19^. DLS could also be used to monitor NP degradation in liquid suspensions, however this approach is severely hampered by the presence of multimodal or highly polydisperse particle distributions, which represent the majority of the tested samples^12^. An alternative method has been introduced more recently with the Nanoparticle Tracking Analysis (NTA)^20^. It relies again on the measurement of the hydrodynamic radius, but this is recovered from sequences of images of single particles. To date, a real time monitoring of NPs in suspension is still an unresolved challenge.

Here we present an in-line optical technique overcoming the limitations typical of DLS and allowing for the efficient characterization of NPs polydispersity, index of refraction and degradation dynamics. Our approach is based on the analysis of the scattered fields^21^ from single NP with an experimental method called Single Particle Extinction and Scattering (SPES)^22^. As a model system we have chosen Poly(D,L-lactide-co-glycolide) nanoparticles (PLGA NPs) because of their clinical relevance and the possibility to finely tune the particle size and drug payloads^6^,^23^. To this purpose, the PLGA NPs were prepared in solution with two different molecular architectures (homogeneous matrix or core-shell structure) encapsulating either a hydrophobic or hydrophilic model drug, respectively.

## Results

**Figure 1:**
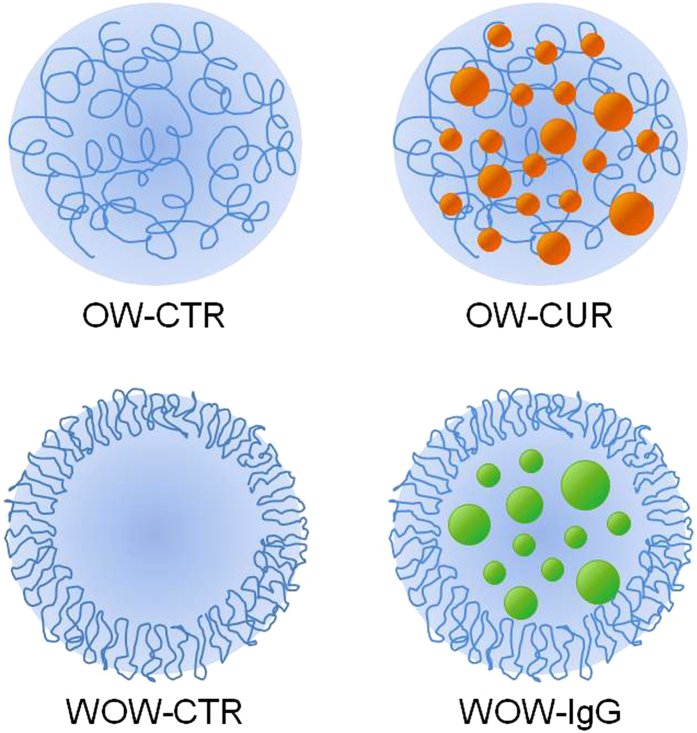
PLGA NPs with different architectures and drug payloads were synthesized. Namely, PLGA NPs consisting of either a homogeneous PLGA/curcumin matrix (OW-CUR) or a core-shell structure encapsulating a model fluorescent protein (WOW-IgG) were studied. Control samples were also prepared (OW-CTR and WOW-CTR).

Two different types of PLGA nanoparticles (PLGA NPs) were synthesized by either Oil-in-Water (OW) or Water-in-Oil-in-Water (WOW) solvent evaporation emulsion techniques. In the OW process, curcumin as hydrophobic drug was encapsulated into the PLGA NPs and a homogeneous polymer/curcumin matrix was obtained (OW-CUR). The WOW synthesis was selected to encapsulate a model fluorescent protein (goat anti-mouse IgG1 labelled with Alexa Fluor® 488, IgG1-488) and resulted in core-shell nanoparticles (WOW-IgG). As a control, both the syntheses were run in the absence of curcumin or IgG1-488. The structures of PLGA NPs are schematically represented in Fig. 1.

Immediately after synthesis, the PLGA NPs were characterized by confocal microscopy in order to assess the encapsulation of either curcumin or IgG1-488 in the PLGA matrix (see method section for detailed description of the characterization procedure). Confocal microscopy results confirmed the co-localization of particle fluorescence and scattered transmitted light, suggesting that either curcumin or IgG1-488 were encapsulated into the PLGA NPs (Supplementary material). Samples morphology was assessed by Scanning Electron Microscopy (SEM) analysis. PLGA NPs was found to be round shaped in all the tested samples, as shown in the Supplementary material.

In order to characterize the size distribution of the NPs as well as their structural evolution in suspension, we applied the standard approach based on DLS. DLS is based on the statistical analysis of the fluctuations of the light scattered at 90° by a collection of particles suspended in a liquid. Brownian motions give rise to a randomly fluctuating intensity signal, whose correlation time is related to the Stokes-Einstein diffusion coefficient of the particles. The statistical analysis of the intensity fluctuations gives the average hydrodynamic radius of the particles^24^. The signal is generated by a number of particles, so that the information about the radius is statistical. Proper, refined analysis tools can access to the polydispersity of the size distribution and, in some cases, to its kurtosis. DLS therefore accesses the statistical properties of the particle motions in the liquid and it exploits the scattering of light to generate the needed signals containing the information. In a similar way, NTA relies on the collection of 90° scattered light to generate images of single particles. Sequences of images permit to recover again the diffusion coefficient, overcoming the DLS limitation imposed by the simultaneous measure of a number of scatterers.

**Figure 2:**
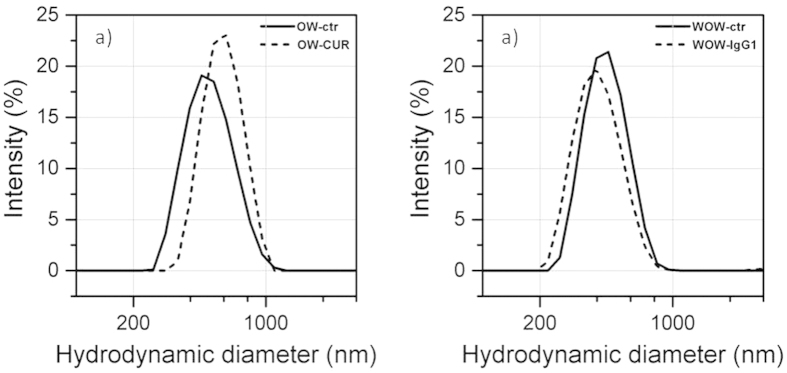
The size profile of the tested PLGA NPs obtained by DLS analysis. The intensity of scattered light is plotted as a function of the hydrodynamic diameter. The Z-average (Z-ave) values of the tested NPs were the following: 594.4 nm (OW-CUR), 521.9 nm (OW-CTR), 475.3 nm (WOW-IgG), 491.6 nm (WOW-CTR). The polydispersity index (pdI) was reported to be 0.127 (OW-CUR), 0.188 (OW-CTR), 0.310 (WOW-IgG), 0.264 (WOW-CTR).

The PLGA NPs were suspended in MilliQ water and characterized by a Zetasizer Nano ZS90 (Malvern Instruments) to obtain the size distribution as hydrodynamic diameter. Results are shown in Fig. 2. The plots represent the size distributions described by the average diameter, and the standard deviation, which indicates the polydipersity^25^. The curcumin-loaded PLGA NPs (OW-CUR) exhibited larger size with respect to the control (OW-ctr), thus suggesting that the model drug was effectively encapsulated in the polymeric matrix. Further, the polydispersity index was low for both samples, indicating a very narrow size distribution. The WOW synthesis resulted in NPs having smaller size and higher Polydispersion Index (PdI) than samples obtained by OW process. This is possibly due to the fact that ultrasound energy was applied to the systems twice. Although DLS analysis was able to show differences in mean size between PLGA NPs encapsulating either curcumin or IgG1-488 and their negative controls, no qualitative changes in terms of composition and polymer degradation were appreciated by this technique (Fig. 2).

The behavior of polymeric nanoparticles in solution is a very important parameter affecting their performances as drug carriers and their toxicity. Here, we have used DLS to characterize the structure evolution of PLGA NPs in phosphate buffered saline (PBS, pH 7.4). Samples were incubated at 37 °C under constant rotation and characterized at determined time points. To assess the nanoparticles stability by DLS, several parameters including the scattering intensity (count rate), mean size and correlogram shape were considered. Results are presented in the Supplementary material.

**Figure 3:**
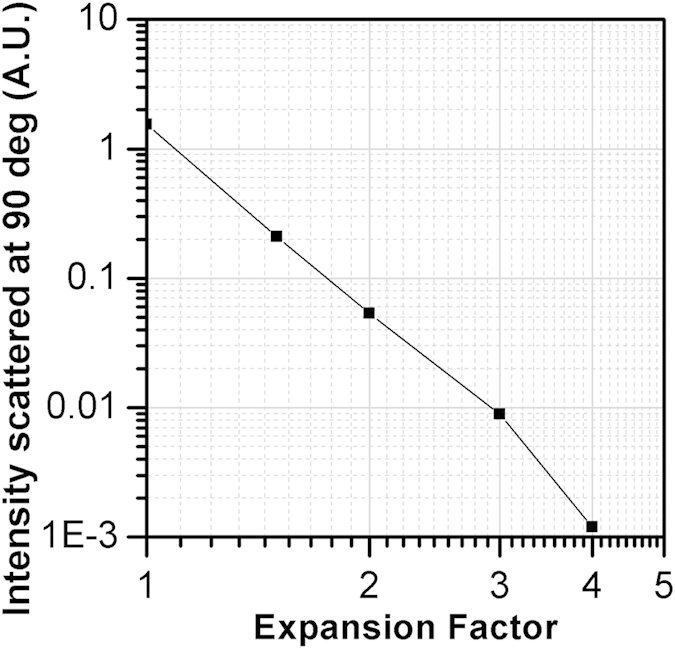
Results of simulations describing intensity (in arbitrary units) of the signal expected for DLS measurements at 90° as a function of the size increase, here expressed as the expansion factor of the particle diameter.

After 24 hours of incubation DLS measurements were almost impossible due to a dramatic decrease in signal intensity. It is widely known that PLGA NPs degrade in aqueous suspension by cleavage of their ester bonds causing the swelling and size growth of NPs. This has a consequence the narrowing of the light scattering lobe in the forward direction, as well as the reduction of the relative NPs refractive index, which reduces the total amount of the scattered light. Both these effects are detrimental for DLS, causing a significant decrease in the scattered intensity signal. To confirm this model, we report in Fig. 3 the numerical simulation results of the scattered intensity upon NPs degradation. A collection of NPs with a distribution of sizes ranging from 0.1 up to 1 μm in diameter, equally distributed has been assumed to be composed of pure PLGA at the initial stage. Swelling has been assumed to give rise to size growth by factors 1.5, 2, 3, 4 of each particle of the distribution. Simulations were performed with the Amsterdam Discrete Dipole Approximation code^26^,^27^ as detailed in the Supplementary material.

The simulation results reported in Fig. 3 clearly show that the intensity detected using the DLS technique decreases by orders of magnitude during degradation of the NPs in solution, preventing the DLS ability to follow the NPs evolution and modification. This is expected due to the decrease of the relative refractive index induced by the swelling process. Notice that a very similar limitation is imposed to the amount of light collected with the NTA to generate the image of a single particle.

**Figure 4:**
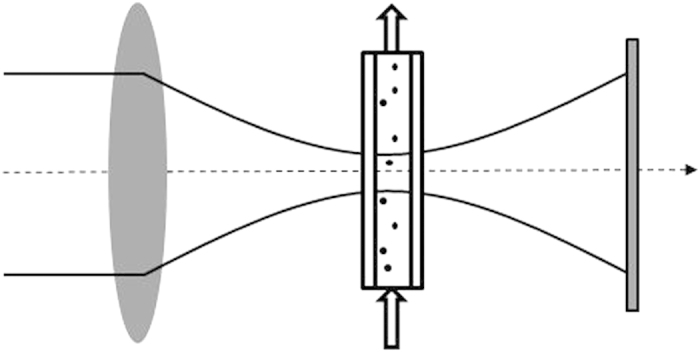
Schematic representation of the SPES apparatus. A laser beam is focused by a lens into a flow cell, through which the sample is driven at constant velocity. The light emerging from the cell, composed by the intense transmitted and the faint scattered fields, superimpose on the sensor.

In order to overcome the severe limitation of DLS as an in-line characterization approach, we propose and demonstrate the use of SPES method to characterize the relevant physical properties of NPs in solution and their evolution. In the SPES method, exploiting a very simple optical layout, single particles in a uniform, laminar flow are driven at a given speed through a tightly focused laser beam (Fig. 4). The superposition of the faint scattered and the intense transmitted fields gives rise to time dependent interference patterns. Intensity modulations are therefore proportional to the amplitude of the forward scattered field, while the power reduction of the entire beam, which rigorously gives the total extinction cross section of the particle, provides information enough to a complete characterization of the scattered field^28^,^21^. A schematic representation of the SPES experimental apparatus is shown in Fig. 4. A lens focuses the light of a laser beam into a flow cell, through which the sample is driven at constant speed. The transmitted beam and the forward scattered wave superimpose and give rise to a time dependent interference pattern collected by a segmented photodetector which generates electrical signals. A dedicated data analysis then recovers the basic features of the complex scattered field, so that the SPES method can be employed to realize the complex field approach recently proposed^21^ to precisely determine two independent parameters for each particle, the *extinction cross section* and the *optical thickness* of the particle, *i.e.* the product *s* = *d (m−1)*, where *d* is the particle diameter and *m* is the refractive index relative to the surrounding medium. Especially for spherical nanoparticles, from the combination of the two measured parameters an estimate of the average refractive index is accessible, and in turn the measure of the actual particle size is obtained with a better accuracy compared to traditional scattering methods as reported in a previous work^22^. Here we focus on the refractive index. We show here that by taking advantage of the measurement of many polydisperse spherical particles, the sensibility of the method to differences of refractive indexes increases enough to detect minute changes both in the nanoparticle structure and composition. Notably, this analysis can be run even if the refractive index is not precisely known *a priori*^22^. Notice that just polydispersity is one of the typical features of real samples, and also the main limiting element for traditional methods. Measuring a sample requires some minutes to accumulate a number of events large enough to limit uncertainties in the measurements of the RID and PSD. Many 10^3^ or some 10^4^ events are collected for each measurement in the data presented here. Thanks to the single particle measurements, the repeatability of the results is just determined by the number of measured particles in each bin simply because of Poisson’s statistics. More details of the method can be found in literature^21^,^22^.

PLGA NPs samples were characterized by SPES in terms of the refractive index distribution (RID) and the particle size distribution (PSD). We focused our analysis on the average relative refractive index *m* over a given, extended size range, approximately from 200 to 1000 nm.

Since the WOW-IgG and WOW-ctr samples did not show any significant difference in terms of RID and PSD, the WOW-ctr was used a reference for comparison with OW-ctr and OW CUR.

**Figure 5:**
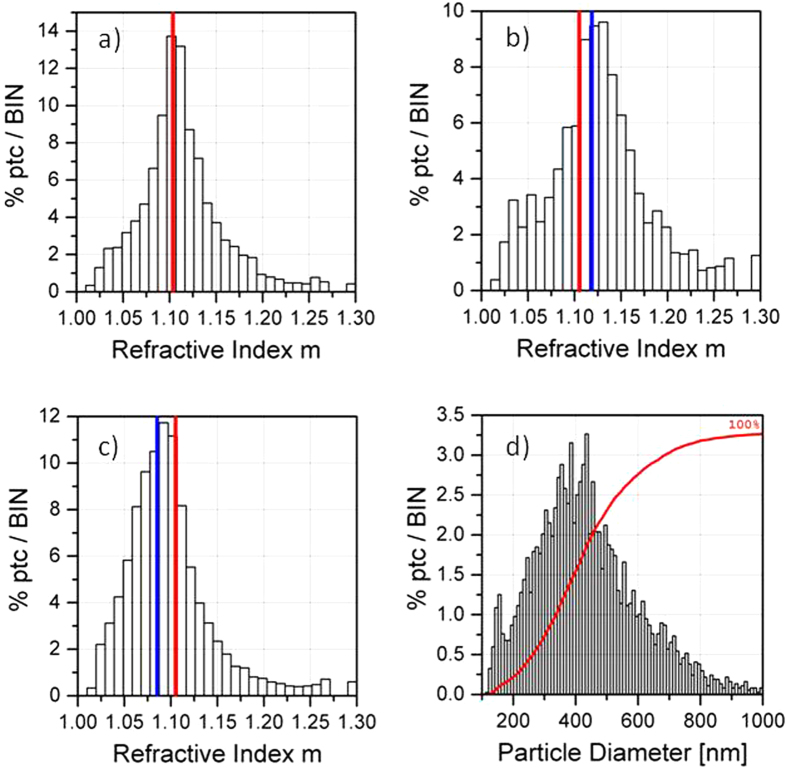
(**a**–**c**) the RIDs for the samples considered here (see text for details). (**d**) the PSD obtained for the sample in a) is shown, which is almost identical to those obtained for (**b**,**c**) (see Supplementary Figure 5).

In Fig. 5 we report the RID of the OW-ctr, with the red line indicating the median value of the distribution, *m* = 1.105. We notice that the RID is almost symmetric about the red line, except for a negligible tail at the lowest values. In Fig. 5b,c we report the RIDs for the OW-CUR and WOW-ctr respectively. The blue lines indicate the median values of the two distributions, *m* = 1.12 and *m* = 1.085, whereas the red lines indicate the same median value shown in Fig. 5a. In Fig. 5d we plot an example of the PSD, here obtained from the data corresponding to the RID shown in a). Since all the PSDs are extremely similar, we plot just the one in d) for the sake of shortness. PSDs corresponding to the remaining two samples are reported in the Supplementary material.

Besides the average value of the refractive indexes, one can notice that in Fig. 5b the RID is still symmetric about the blue line within a good approximation, as in Fig. 5a for OW-ctr. Moreover, the width of the distribution is not increased by an amount comparable with the shift in the average refractive index. This supports the idea that i) all the NPs are loaded with curcumin, meaning that NPs like OW-ctr are not present in the suspension; ii) curcumin is almost uniformly distributed in all the NPs. This result is fully compatible with the oil-in-water emulsion method adopted for NPs synthesis, in which curcumin is homogeneously mixed in the organic phase. As a direct, independent check, we have analyzed these samples at the confocal microscope, which confirms this result (see the supporting material). On the contrary, in Fig. 5c the RID is shifted in the opposite direction, accordingly with the presence of water inside the NPs that reduces the polarizability of the whole object, determining a reduction of the effective refractive index *m*. Noteworthy, a pronounced skewness is evident here, showing that the refractive indices are spread over a more extended range. This suggests a distribution of water volumes encapsulated within the NPs. Using the refractive index we can recover the size of each particle^21^. Notice that since SPES measures single particles, while DLS relies on the signal due to a huge number of particles, the size recovered in our case is intrinsically more reliable. From the point of view of the PSD, although very similar, slight differences are found. The mode values are respectively a) 476 nm, b) 496 nm and c) 456 nm. Differently, DLS data provided only vague indications on structural changes of the PLGA NPs after encapsulation of either curcumin or IgG-488. Indeed, the overall size of OW-CUR and WOW-IgG was found to be respectively larger and smaller with respect to control experiments (OW-CTR and WOW-CTR). Notably, to interpret the DLS results, comparison with control experiments as well as previous experimental expertise were required.

In order to compare the SPES performances in terms of characterization of the PLGA NPs modification and degradation in solution, we performed the analysis of NPs in PBS in the same conditions used in the case of DLS analysis.

**Figure 6:**
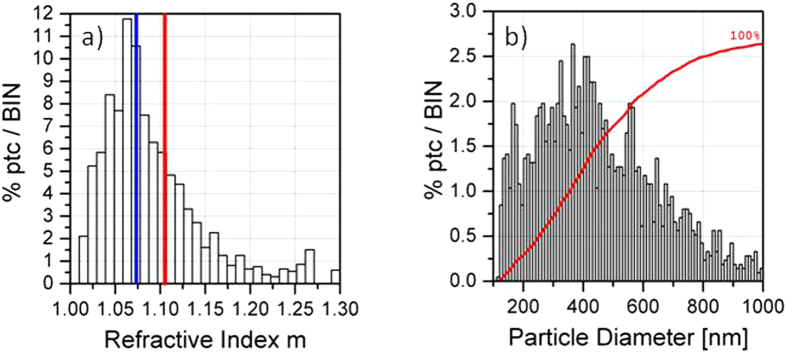
Results obtained for the degradation of OW-ctr sample with added PBS, measured after 24 h incubation at 37 °C. (**a**)RID, (**b**) PSD.

Figure 6 shows the effects of degradation of PLGA NPs suspended in PBS as detected by SPES. Panel a) represents the RID obtained from data collected after incubation, which shows an average value (blue line) appreciably smaller than that measured before incubation (red line), and again the evident skewness indicates a population of particles with different degrees of compactness. In this case the PSD represented in b) slightly changes with respect to the OW-ctr case in Fig. 5a), the mode increasing up to 515 nm.

In contrast with DLS, the SPES method maintains a good sensibility to the swelled particles thanks to the peculiar forward scattering scheme adopted here. As pointed out above for the DLS case, swelling causes particles to be larger and larger, endowed with smaller and smaller relative refractive indexes. As a consequence the total amount of scattered light decreases upon swelling, and the scattering lobe becomes narrower. At variance with the DLS case, where both effects cause a decrease of the detected signals, in SPES they compete and approximately compensate each other thanks to the peculiar forward scattering scheme.

**Figure 7:**
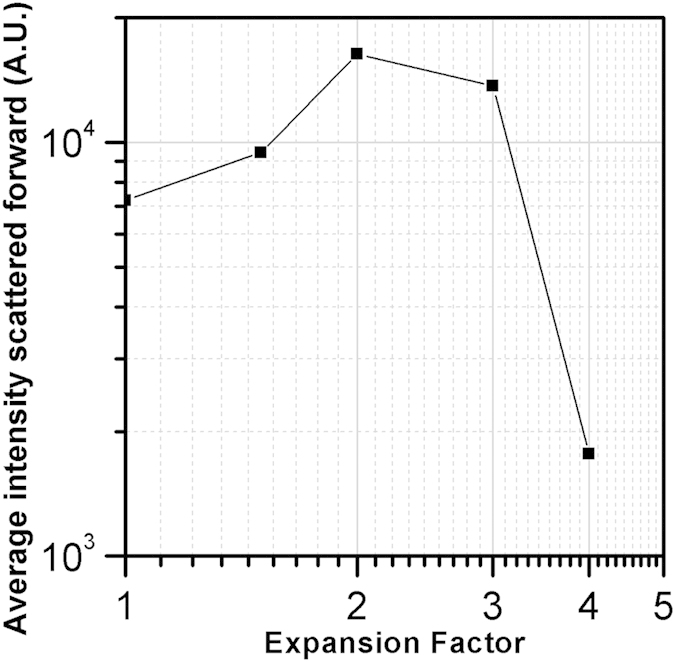
Average intensity scattered forward from the same particles considered in Fig. 3 upon swelling by the factors reported on the abscissas.

In order to provide a quantitative description of this effect, we consider the same collection of particles adopted above to study the DLS signal decrease, in such a way to obtain a fully coherent comparison between the two methods. In Fig. 7 we report the results of numerical simulations of the average light intensity scattered forward by single NPs undergoing swelling. Notice that the intensity changes determined by the degradation of the NPs are negligible until the size increases by a factor 4. This is completely different to what we have obtained for exactly the same particles we have considered in Fig. 3 for the 90° light collection (DLS and NTA), where the signals decrease by orders of magnitude during the degradation process.

## Conclusions

We demonstrated the capability of a very simple and straightforward optical technique based on the measurement of single particle optical extinction and scattering for the characterization of the structural and functional properties of polymeric nanoparticles in solution. In particular we showed that the SPES method provides an effective characterization of single NPs in terms of both size and refractive index. This is of particular importance when polydisperse suspensions are considered, that is when typical DLS data are affected by statistical effects due to the simultaneous presence of a number of scatterers in the beam. SPES takes huge advantage from the single particle detection, which prevents from the use of any inversion algorithm for extracting information about the sample.

We characterized PLGA NPs with different payloads and under different conditions showing that SPES is capable to provide quantitative and detailed information that are out of reach of the traditional DLS approach. Another advantage of the SPES method is the possibility to operate in-line. This is of paramount importance in view of the development of pharmaceutical industrial processes for NPs requiring process analytical technologies to assure quality and safety.

## Methods

### Materials

Poly(Lactide-co-Glycolide) acid (PLGA) with a 50:50 copolymer ratio and free ester end groups (Resomer® RG 504, MW 38,000–54,000), Poly(Vinyl) Alcohol (PVA, Mowiol® 4–88 MW 31,000), D-mannitol, D-(+)-trehalose dehydrate, curcumin and ethyl acetate were obtained from Sigma Aldrich and used without further purification.

Alexa Fluor® 488 goat anti-mouse IgG1 (isoform γ1, IgG1-488) was purchased from Life Technologies and diluted up to 0.3 mg/mL in a medium containing 0.1 M sodium phosphate, 0.1 M NaCl and 5.0 mM sodium azide (Sigma Aldrich). Bidistilled water (MilliQ, Millipore) was used in all the experiments.

### PLGA nanoparticles synthesis

To obtain PLGA NPs encapsulating curcumin (OW-CUR sample), curcumin and PLGA were dissolved in ethyl acetate at the concentration of 5.0 and 50.0 mg/mL, respectively. The curcumin-PLGA solution was emulsified with 1.0 mL of PVA 0.2% w/v by ultrasound (Vibracell VCX130, 30% amplitude, 15 seconds) and the obtained emulsion was diluted with 50 mL of PVA 0.3% w/v. Then, the organic solvent was evaporated via magnetic stirring for 3.5 hours. Nanoparticles were collected and washed with three cycles of centrifugation at 15,000 g for 20 minutes. As a control (OW-ctr), the same procedure was performed without adding the model drug.

Differently, the PLGA NPs containing the model protein (WOW-IgG sample) were prepared by a two steps procedure. First, an aqueous solution of IgG1-488 (25 μL, 0.3 mg/mL) was added to the PLGA/ethyl acetate solution (50.0 mg/mL) and sonicated at 30% amplitude for 15 seconds, to obtain the primary water-in-oil emulsion. This emulsion was then added to 1.0 mL of PVA 0.2% w/v and sonicated using the same protocol described above, to achieve the multiple water-in-oil-in-water (WOW) emulsion. Finally, the WOW emulsion was diluted in the dispersing phase (50.0 mL PVA 0.3% w/v) and the organic solvent was evaporated via magnetic stirring for 3.5 hours. Control PLGA NPs (WOW-ctr) were prepared according to the same procedure described above. In this case, instead of the IgG1-488 solution, the inner core was composed by a control medium (0.1 M sodium phosphate, 0.1 M NaCl and 5.0 mM sodium azide).

Nanoparticles were washed by three cycles of centrifugation (15,000 g, 20 minutes) to remove excess surfactant and non.-encapsulated IgG.

### PLGA nanoparticles characterization

Immediately after synthesis, the PLGA NPs were characterized by Dynamic Light Scattering (DLS) to assess their size and surface charge. To this aim, the PLGA NPs pellet was diluted 1:1000 in MilliQ water and characterized by a Nano ZS90 (Malvern Instruments).

The same samples were then diluted 1:500 for the dimensional and qualitative characterization with SPES. To investigate the encapsulation of the model drugs as well as the morphology of the PLGA NPs, analyses were performed by confocal and scanning electron microscopies, respectively. Before analyses, the NPs pellet was resuspended in MilliQ water (1:200) and dialyzed for three hours using a Spectrapor membrane (cut off 100 KDa). The purified suspensions were additionally diluted 1:500 and 5.0 μL of the final samples were left drying on a glass slide for confocal microscopy (Leica TCS SP5 AOBS, Leica Microsystems GmbH) or on a silicon support for Scanning Electron Microscopy (SEM, Zeiss Sigma, Carl Zeiss NTS GmbH) analyses.

The confocal analysis of the OW-CUR and OW-ctr samples was performed by excitation with the 458 nm argon laser line passed through an objective lens 63x, oil immersion, numerical aperture 1.4. Similar conditions were applied to analyse the WOW-IgG and WOW-ctr samples, except the excitation wavelength (argon-laser light of 488 nm).

Before the SEM analysis, samples were left drying in a vacuum pump for 2 h. All of the SEM images were acquired with an accelerating voltage of 1 kV. Dimensional distribution of PLGA NPs was calculated, by meaning of the open source software Image J.

### Degradation studies

Before performing the degradation studies, each PLGA NPs batch was added with 1% (w/v) D-mannitol as cryoprotectant and lyophilized. Degradation was monitored by resuspending the PLGA NPs in phosphate buffered saline (PBS) at physiological pH (7.4) and keeping the samples at 37 °C under constant rotation. At determined time points the suspensions were characterized by DLS without further dilution, whereas analysis by SPES was run after 1:500 dilution in PBS.

## Additional Information

**How to cite this article**: Potenza, M. A. C. *et al.* Single particle optical extinction and scattering allows real time quantitative characterization of drug payload and degradation of polymeric nanoparticles. *Sci. Rep.* **5**, 18228; doi: 10.1038/srep18228 (2015).

## Supplementary Material

Supplementary Information
