# Supplementary material for: Single particle optical extinction and scattering allows real time quantitative characterization of drug payload and degradation of polymeric nanoparticles
Source: Sci Rep. 2015 Dec 15;5:18228. doi: 10.1038/srep18228 (PMC4678328; doi:10.1038/srep18228)
Supplement: Supplementary Information [file srep18228-s1.doc]

Single particle optical extinction and scattering allows real time quantitative characterization of drug payload and degradation of polymeric micro- and nanoparticles

M.A.C. Potenza, T. Sanvito, S. Argentiere, C. Cella, B. Paroli, C. Lenardi, P. Milani


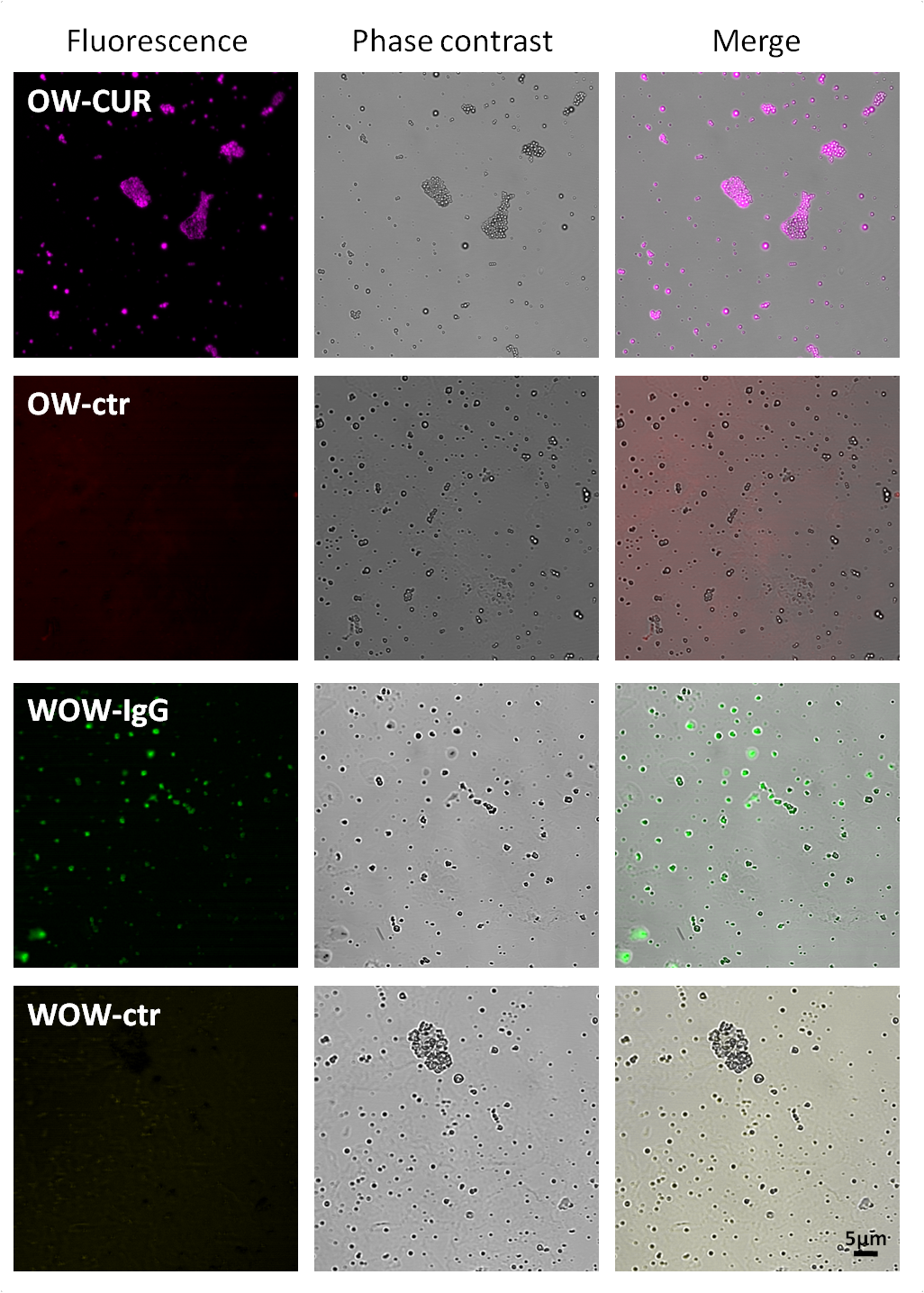


Supplementary Figure 1. Confocal microscope analyses. By merging (panels on the right) the phase contrast (central panels) and the fluorescent channels images (panels on the left), it was possible to note that almost all the nanoparticles with curcumin (OW-CUR) or IgG1_Alexa488 (WOW-IgG) actually encapsulated the fluorophore, while the respective controls (OW-ctr and WOW-ctr) did not have fluorescence.

Supplementary Figure 2. Scanning Electron Microscopy (SEM, Zeiss Sigma) characterization. For each synthesis, two different magnifications were reported.

Supplementary Figure 3. Distribution analysis from SEM images. By means of the open-souce software Image J, it was possible to further analyze SEM images and additionally characterize NPs distributions.

Supplementary Figure 4. Particle size distributions obtained by DLS analysis at different time points. In samples obtained by double emulsion (WOW-ctr and WOW-IgG), degradation was complete after 24 hr, therefore time points 48 hr and 72 hr are not reported.

Supplementary Table 1. Main parameters obtained from DLS analysis at different time points. In samples obtained by double emulsion (WOW-ctr and WOW-IgG), degradation was complete after 24 hr, therefore time points 48 hr and 72 hr are not reported.


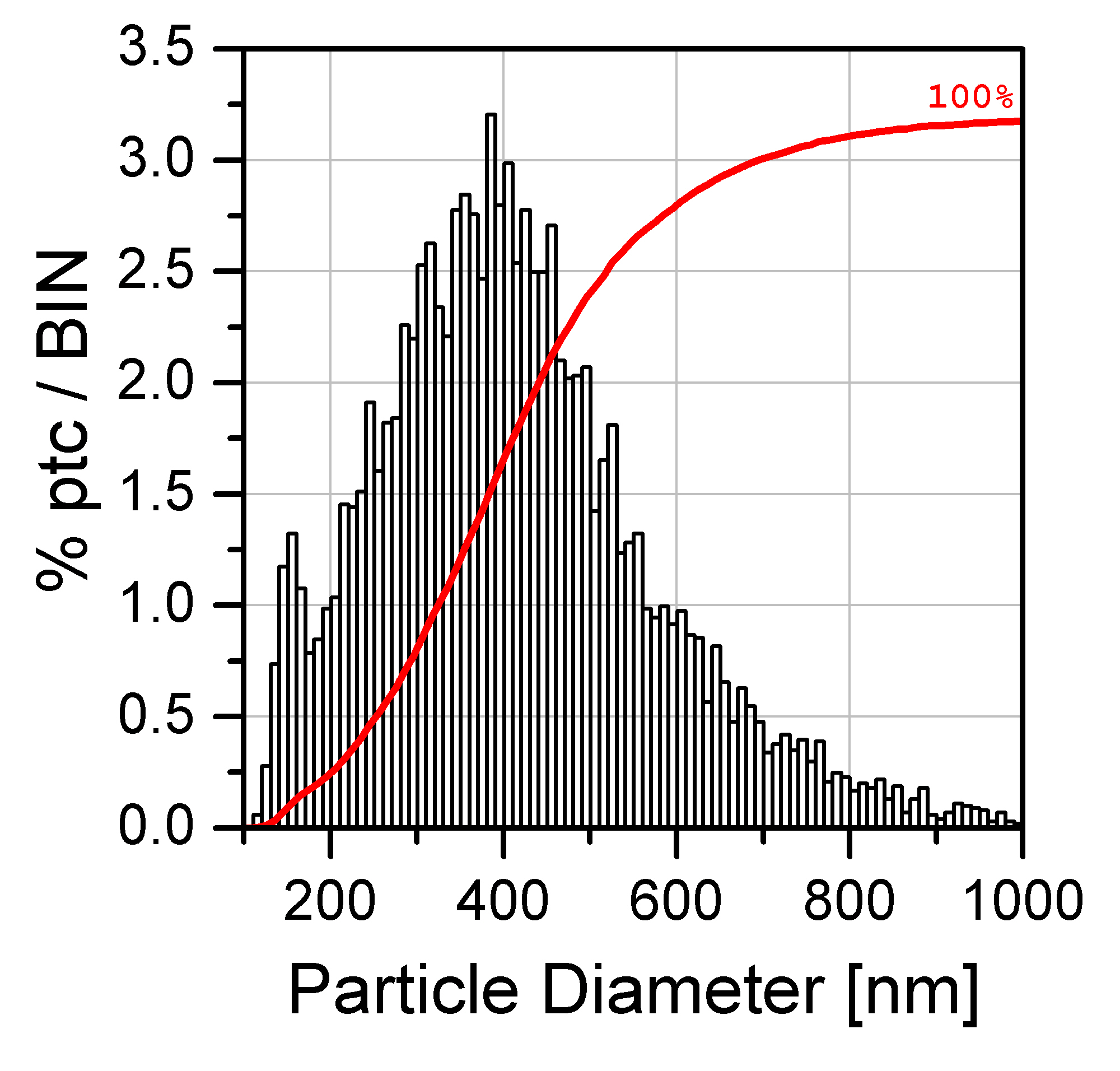

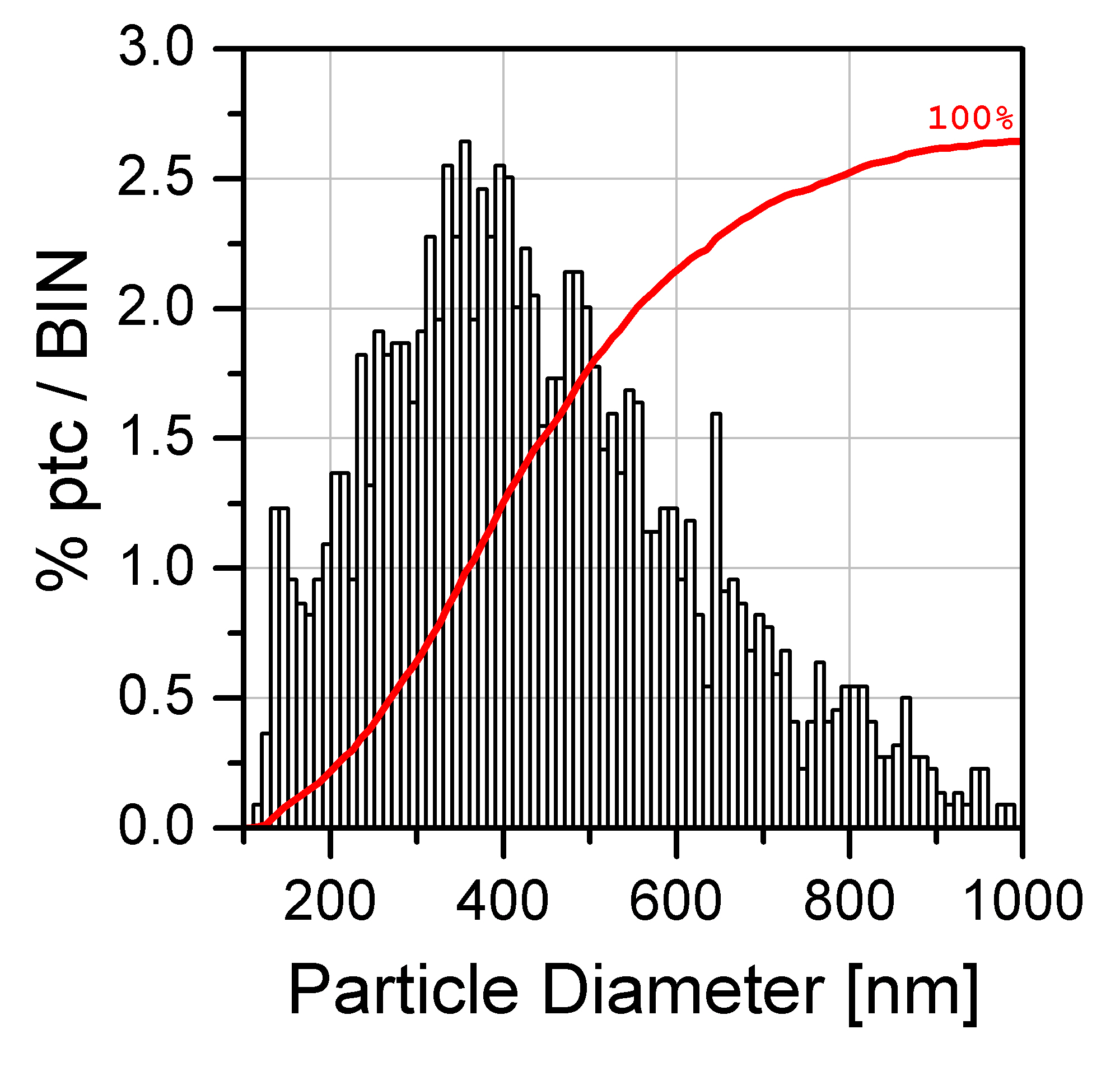


Supplementary Figure 5. Particle size distributions obtained from the SPES data for the two samples reported in Fig. 5 b) and c) respectively.

Simulation of the swelling process

We briefly describe the numerical simulations performed to describe the effect of swelling.

We consider a collection of NPs with a distribution of sizes from 0.1 up to 1 µm in diameter, equally distributed, composed of pure PLGA (refractive index 1.47 in vacuum). Swelling is described by assuming that the suspending liquid uniformly penetrates the NPs, thus increasing the volume proportionally to the water volume. For the sake of simplicity, we will assume that the amount of material composing each cluster is constant with time. Spherical shape is assumed during the overall process, so to prevent spurious scattering effects due to shape and orientation. Growth factors of 1.5, 2, 3, 4 times the diameter are considered for each particle in the distribution. Again, this choice make the result independent from any PSD effect.

When a particle increases in size the refractive index decreases due to the penetration of water. The particle is then described by a sphere with a refractive index obtained through the Lorentz-Lorenz relation by assuming a uniform mixing of PLGA and water accordingly to the amount of water imposed by the size growth.

We simulated single particle scattering through the well established Amsterdam Discrete Dipole Approximation code^25,26^, and averaged the results for any given growth factor. The intensity reported in Fig. 3 is obtained by averaging over a solid angle wide enough to minimize the oscillations due to Mie resonances. In Fig. 7 the forward scattered intensity is just the zero angle intensity.
